# Peritoneal dialysis related peritonitis: insights from a long-term analysis of an Italian center

**DOI:** 10.1186/s12882-024-03594-y

**Published:** 2024-05-11

**Authors:** Luca Nardelli, Antonio Scalamogna, Federico Ponzano, Anna Sikharulidze, Federica Tripodi, Simone Vettoretti, Carlo Alfieri, Giuseppe Castellano

**Affiliations:** 1 Division of Nephrology, Dialysis and Kidney Transplantation, Department of Clinical Sciences and Community Health, Fondazione IRCCS Ca’ Granda Ospedale Maggiore Policlinico 1, Via della Commenda 15, Milano, 20122 Italia; 2 Department of Clinical Sciences and Community Health, Università degli studi di Milano, Milan, Italy

**Keywords:** Peritoneal dialysis, Peritonitis, Catheter, Mortality, Hospitalization, HD transfer

## Abstract

**Background:**

Peritonitis is a common and severe complication of peritoneal dialysis (PD). For comparative analysis standardized definitions as well as measurements and outcomes are crucial. However, most PD-related peritonitis studies have been using heterogenous definitions and variable methods to measure outcomes. The ISPD 2022 guidelines have revised and clarified numerous definitions and proposed new peritonitis categories and outcomes.

**Methods:**

Between 1st January 2009 and 31st May 2023, 267 patients who started PD at our institution were included in the study. All PD-related peritonitis episodes that occurred in our unit during the study period were collected. The new definitions and outcomes of ISPD 2022 recommendations were employed.

**Results:**

The overall peritonitis rate was 0.25 episode/patient year. Patient cumulative probability of remaining peritonitis-free at one year was 84.2%. The medical cure and refractory peritonitis rates were equal to 70.3 and 22.4%, respectively. Culture-negative peritonitis accounted for 25.6% of all specimens. The rates of peritonitis associated death, hemodialysis transfer, catheter removal and hospitalization were 6.8%, 18.3%, 18.7% and 64.4%, respectively. Relapsing, repeat, recurrent and enteric peritonitis accounted for 7.8%, 6.8%, 4.1% and 2.7% of all episodes, respectively. Catheter insertion, catheter related and pre-PD peritonitis were 4.2, 2.1 and 0.5%.

**Conclusions:**

The implementation of PD-related peritonitis reports using standardized definitions and outcome measurements is of paramount importance to enhance clinical practice and to allow comparative studies.

## Introduction

Peritoneal dialysis (PD)-related peritonitis represents the leading cause of permanent transfer to hemodialysis (HD) [1, 2]. Its occurrence is strongly associated with an increased risk of hospitalization, death as well as higher healthcare costs and long-term peritoneal membrane disfunction [3–5]. Standardization of peritonitis definitions, measures and outcomes is crucial to allow comparative analysis of interventions and delineate benchmarks of performance. In fact, most PD-related peritonitis studies have been using heterogenous definitions as well as variable methods to report and measure outcomes [6, 7].

According to the latest International Society of Peritoneal Dialysis guidelines (ISPD), PD-related peritonitis can be further classified according to cause, association with catheter implantations, exit-site/tunnel infections, timing in relation to previous episodes and outcomes [8].

Thus, deeper comprehension concerning the incidence, prevalence, and outcome of cause- and time specific peritonitis in contemporary cohorts is ncessary to develop peritonitis prevention strategies and enhance its treatment.

In addition, to improve and address practice variations on peritonitis prevention strategies and promote its treatment, a better comprehension of the peritonitis causes is also required.

## Methods

### Participants and study design

This is a single center study. Adults (> 18 years) who started PD between January 1st 2009 and May 31st 2023 at Fondazione IRCCS Ca’ Granda Ospedale Maggiore Policlinico were included. Data were collected retrospectively from clinical records at our unit by reviewing an electronic database (Galenus®, Infogramma s.r.l., Milan, Italy). All patients continued the follow up to the end of the study period, or until the discontinuation of PD due to death, kidney transplantation, transfer to HD or to another PD center.

The peritonitis rates (episodes per patient year) were calculated as the total number of peritonitis episodes divided by the number of patient–years on PD. In this calculation relapsing episodes were not considered. Culture-negative, pre-PD, PD catheter insertion-related, repeat, recurrent and relapsing peritonitis as well as catheter removal, hemodialysis transfer and death associated to peritonitis were reported as percentage of all peritonitis episodes.

### Definitions

Peritonitis was diagnosed when at least two of the following conditions were present: (1) clinical features consistent with peritonitis; (2) dialysis effluent white blood cell count (WBC) > 100/µL or > 0.1 × 109/L (after a dwell time of at least 2 h), with > 50% polymorphonuclear cells; and (3) positive dialysis effluent culture [8].

Culture-negative peritonitis was defined when peritonitis was diagnosed using the previous criteria, but without microorganism identification on culture of dialysis effluent. Enteric peritonitis was defined as peritonitis arising from an intestinal source. Pre-PD peritonitis was defined as a peritonitis episode occurring after PD catheter insertion and prior to commencement of PD treatment (first PD exchange execution), while for PD-related peritonitis (after PD commencement) time at risk started from the day of PD initiation. Peritoneal dialysis catheter insertion-related peritonitis was defined as an episode of peritonitis that occurred within 30 days of PD catheter insertion.

Exit-site infection was defined as the presence of purulent discharge at the catheter epidermal interface, while tunnel infection was diagnosed in the presence of clinical inflammation (erythema, swelling, tenderness or induration) along the catheter tunnel.

Catheter-related peritonitis was defined as a peritonitis that occurs in a temporal conjunction (within 3 months) with a catheter infection (either exit-site or tunnel) with the same organism at the exit-site or from a tunnel collection and in the effluent, or one site sterile in the context of antibiotic exposure. Relapsing peritonitis was defined as an episode that occurred within 4 weeks of completion of therapy of a prior episode with the same organism or one culture negative episode; while recurrent peritonitis as an episode that occurred within 4 weeks of completion of therapy of a prior episode but with a different organism. Repeat peritonitis was diagnosed as an episode that occurred more than 4 weeks after completion of therapy of a prior episode with the same organism.

Outcome of peritonitis was classified as medical cure, refractory, peritonitis-associated catheter removal, hemodialysis transfer, death and hospitalization.

Medical cure was defined as a complete resolution of peritonitis together with none of the following complications: [1] relapse/recurrent peritonitis, [2] catheter removal, [3] transfer to hemodialysis for ≥ 30 days or death. Refractory peritonitis was defined as an episode with persistently cloudy dialysate or persistent dialysis WBC > 100 × 109/L after 5 days of an appropriate antibiotic therapy.

Peritonitis-associated catheter removal was defined as a removal of PD catheter as part of the treatment of an active peritonitis episode, while peritonitis-associated hemodialysis transfer as a shift from PD to HD for any period as part of the treatment for a peritonitis episode. Peritonitis-associated death was diagnosed in case of death occurring within 30 days of peritonitis onset, or death during hospitalization due to peritonitis, while peritonitis-associated hospitalization was defined as hospitalization precipitated by the occurrence of peritonitis for the purpose of peritonitis treatment delivery [8].

### Clinical management

In every patient a straight double-cuffed Tenckhoff catheter was placed with a modified double purse string technique around the inner-cuff either in semi-surgical or surgical procedure, as described elsewhere [9, 10]. The creation of the subcutaneous part was achieved by using a piercing tunneller in a direction able to minimize shear forces with the superficial cuff located at least 4 cm from the exit of the skin [11]. As far as routinely exit-site care is concerned, patients were instructed to apply hydrogen peroxide followed by 5% sodium hypochlorite solution to the skin surface three to four times per week. A twin bag disconnect system was the standard connecting system used for continuous ambulatory peritoneal dialysis (CAPD) and a luer lock method was used for automated peritoneal dialysis (APD). Initial antibiotic treatment for peritonitis were intraperitoneal administration of cefazolin (loading dose of 500 mg/L in the first exchange and maintenance dose of 250 mg/L in each subsequent exchange) plus tobramycin (loading dose of 100 mg intravenous and maintenance dose of 8 mg/L in each exchange). Patients treated by APD were temporarily converted to CAPD. Antibiotic regimen was then adjusted according to the results of the culture. Direct WBC/high power field of the peritoneal effluent was routinely performed every day to evaluate the primary response. Patients received effective antibiotic for either two or three weeks based on the causative microorganism as suggested by the most recent ISPD guidelines at that time.

When the peritoneal effluent did not clear up after 5 days of effective antibiotic treatment, Tenckhoff catheter was removed, patient was shifted on HD, and target antibiotic therapy was continued.

However, active monitoring of antibiotic effect longer than 5 days was followed in selected cases when PD effluent WBC count was slowly decreasing towards normal.

### Microbiological investigation

Whenever peritonitis was suspected, microbiological examination was promptly accomplished. Culture of peritoneal dialysis effluent was performed using BacTAlert FA Plus Aerobic bottles (Biomerieux, Inc. Durham NC) incubated in Virtuo automated system for 5 days. Positive bottles were subcultured on Blood agar, MacConkey agar, Mannitol salt agar (incubated 48 h at 37 +/- 1ÆC in aerobic conditions), Chocolate agar (incubated 48 h at 37 +/- 1ÆC in 5% CO2), and Sabouraud agar (incubated 48 h at 32 +/- 1ÆC in aerobic conditions). Species identification was obtained with MALDI-TOF Vitek MS (Biomerieux) and antibiotic susceptibility was determined with Vitek2 automated system (Biomerieux) according to EUCAST criteria. Subsequently, empiric treatment of peritonitis was started.

### Statistical analysis

Normally distributed variables are presented as mean ± standard deviation, while nonparametric data are presented as median with interquartile range. Categorical variables are expressed as frequency and percentage. The parametric continuous variables were compared by the Student t-test; otherwise the Mann–Whitney U-test was used. Fisher’s exact test for 2 × 2 contingency tables and Chi-square analysis for larger table were used to compare the nominal data. All probabilities were 2 tailed, and significance level was set at 0.05 to reject the null hypothesis. Life-table analysis (Kaplan-Meier method) was used to calculate the probability for a patient to be free from peritonitis at a given time. Patients who were transplanted, transferred to HD, lost at the follow-up, or died were censored from the calculation. SPSS version 16.0 [SPSS, Inc, Chicago, IL, USA] or PRISM9 (GraphPad, CA, USA) software package was used for the statistical calculations.

#### Ethical approval and informed consent

Treatments and procedures herein reported were in accordance with the ethical standards of the 1964 Helsinki declaration and its later amendments, or comparable ethical standards. All methods were approved by the ethical committee of Fondazione IRCCS Ca’ Granda Ospedale Maggiore Policlinico (approval No. 4759 − 1837/19). Given the observational and retrospective nature of the study, the ethical committee of Fondazione IRCCS Ca’ Granda Ospedale Maggiore Policlinico waived the need for informed consent.

## Results

### Population characteristics

A total of 267 patients received PD in our unit during the study period (January 1st, 2009, to May 31st, 2023). The demographic and baseline clinical characteristics of the population are shown in Table 1. Most patients were male (66.7%) and mainly treated by CAPD (89.1%) with a mean age of 63.1 ± 16.8 years. Patients were on PD for a mean time of 27.2 (IQR 14.5–52.3) months and were followed up for 813 patient-years.

**Table 1:** Baseline clinical characteristics of two-hundreds and sixty-seven patients treated by peritoneal dialysis. ADPKD = autosomal dominant polycystic kidney disease; BMI = body mass index; CAD = coronary artery disease; CAPD = continuous ambulatory peritoneal dialysis; COPD = chronic obstructive pulmonary disease; CVD = cerebral vascular disease; IQR = interquartile range; mCCI = modified Charlson comorbidity index for peritoneal dialysis patients according to Cho et al.; n = number of patients; PD = peritoneal dialysis; RKF = residual kidney function; SD = standard deviation;

	Patients (*n* = 267)
AGE years [mean ± SD]	63.1 ± 16.8
GENDER male [n(%)]	178 (66.7)
CAPD [n(%)]	238 (89.1)
DIABETES [n(%)]	45 (16.9)
CAD [n(%)]	33 (12.4)
CVD [n(%)]	35 (13.1)
COPD [n(%)]	33 (12.4)
MALIGNANCY [n(%)]	45 (16.9)
HEART FAILURE [n(%)]	47 (17.6)
LIVER DISEASE [n(%)]	31 (11.6)
BMI kg/m2 [median (IQR)]	23.6 (21.1–26.2)
mCCI [median (IQR)]	4 (1–7)
RENAL DISEASE	
Hypertensive nephropathy [n(%)]	77 (28.8)
Glomerulonephritis [(n%)]	54 (20.2)
Diabetic nephropathy [n(%)]	28 (10.5)
ADPKD [n(%)]	19 (7.1)
Multiple myeloma [n(%)]	14 (5.2)
Uknown [n(%)]	45 (16.9)
Others [n(%)]	30 (11.2)
FOLLOW UP TIME months [median (IQR)]	27.2 (14.5–52.3)
RKF ml/min/1.73m2 [mean ± SD]	6.1 ± 3.6
DIURESIS VOLUME ml [mean ± SD]	1254 ± 640
KT/V total [mean ± SD]	2.42 ± 0.69
KT/V urinary [mean ± SD]	1.16 ± 0.71
KT/V peritoneal [mean ± SD]	1.26 ± 0.52

### Overall incidence and clinical outcomes

During the study period 113 patients (42.3%) experienced 219 peritonitis, with 19 episodes of relapsing (7.8% of all peritonitis episodes), 15 of repeat (6.8%) and 9 of recurrent (4.1%) peritonitis. Not considering the relapsing episodes, sixty-two patients experienced only 1 peritonitis, whereas the remaining 51 accounted for the remaining 138 episodes (29 patients, 2 episodes; 12 patients, 3 episodes; 6 patients, 4 episodes; 4 patients, 5 episodes).

The overall peritonitis rate was 0.25 episode/patient year of treatment, while the mean time to first peritonitis was 23.9 ± 23.9 months.

**Fig. 1:**
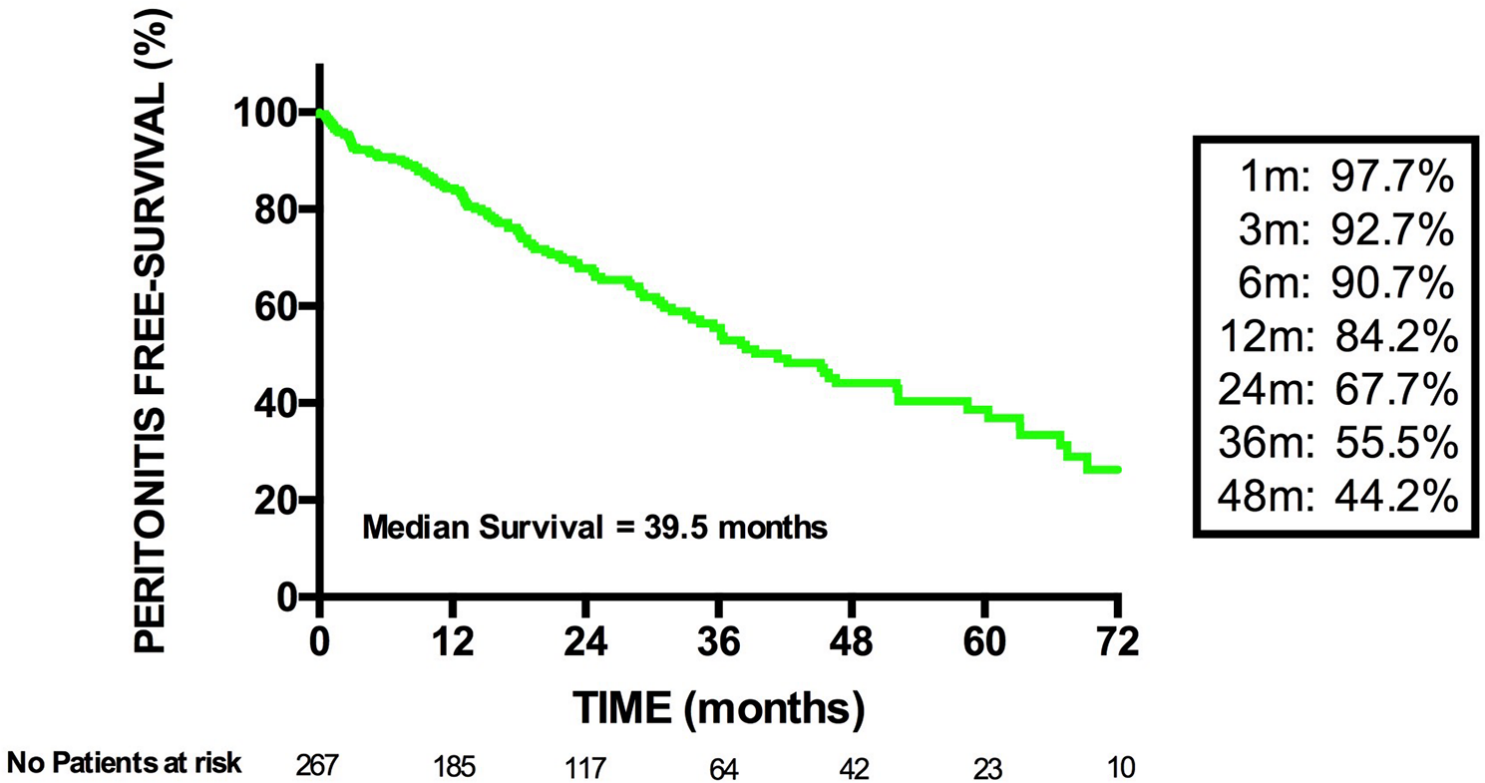
Kaplan-Meier curve of Peritonitis Free-survival. No = number; m = months

Patient cumulative probability of remaining peritonitis-free is reported in Fig. 1. As shown, the percentage of peritonitis-free survival was 97.7, 92.7, 84.2, 67.7, 55.5 and 44.2% at 1, 3, 12, 24, 36 and 48 months, respectively.

**Fig. 2:**
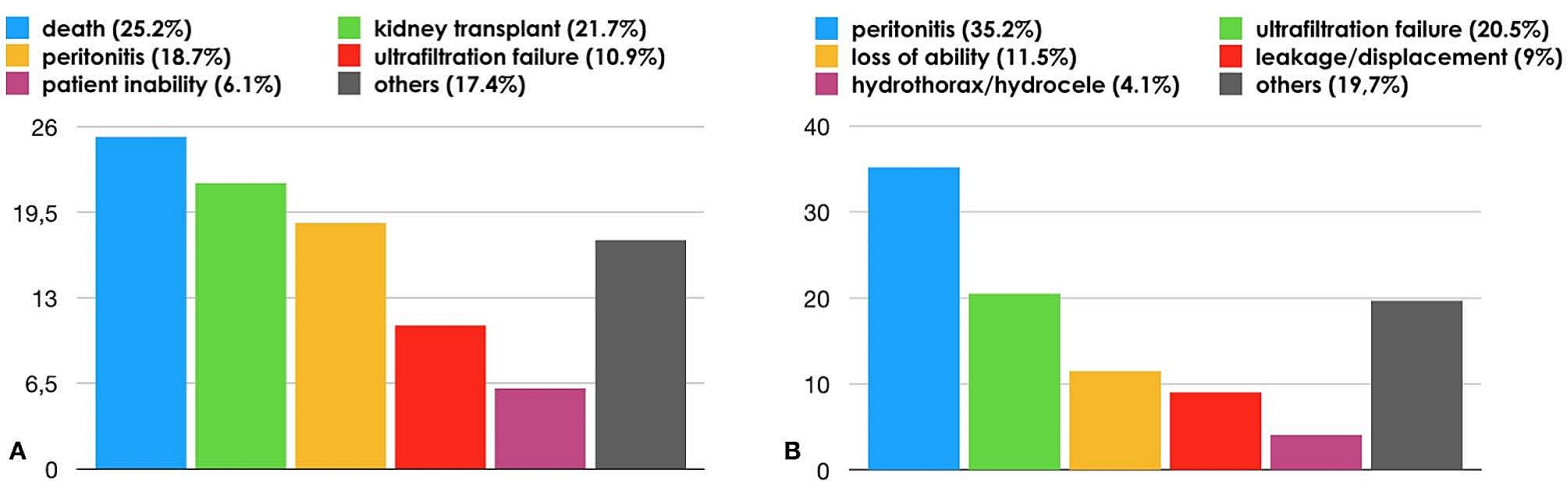
**(A**) causes of peritoneal dialysis discontinuation; **(B**) causes of hemodialysis transfer

During the study period, 230 patients (88.5%) discontinued the dialytic technique; among them 122 were transferred to HD. PD discontinuation and HD transfer causes are summarized in Fig. 2. Not considering death and kidney transplantation, peritonitis accounted for 18.7% of PD termination and represented the main causes of HD transfer (43/122, 35.2%). The main outcomes of PD related peritonitis are summarized in Table 2.

**Table 2:** Measurements and outcomes of peritoneal dialysis related peritonitis. HD = haemodialysis; PD = peritoneal dialysis; PER = peritonitis.

MEASUREMENTS/OUTCOMES	%
Medical cure	70.3
PER-associated hospitalization	64.4
PER-associated catheter removal	18.7
PER-associated HD transfer	18.3
PER-associated death	6.8
Culture-negative peritonitis	25.6
Refractory peritonitis	22.4
Enteric peritonitis	2.7
Relapsing peritonitis	7.8
Recurrent peritonitis	4.1
Repeat peritonitis	6.8
Pre-PD peritonitis	0.5
Catheter insertion-related PER	2.1
Catheter related-peritonitis	4.2

Hospitalization for the treatment of peritonitis was required in 64.4% of the cases (141/219), while the initial antibiotic regimen (cefazolin in association with tobramycin ip) was effective in 131 episodes (141/219, 64.3%). Medical cure was achieved in 70.3% of the cases (154/219). On the other hand, refractory peritonitis accounted for 22.4% of all episodes (49/219), and catheter removal was needed in 18.7% (41/219) of the cases. Since simultaneous removal and replacement of the catheter with immediate restart of PD exchanges was performed in one case of refractory episode, peritonitis associated hemodialysis transfer rate was equal to 18.3% (40/219). Fifteen deaths occurred within 30 days of peritonitis onset, or during hospitalization. Thus, the percentage of peritonitis associated death was 6.8% (15/219).

### Causative organisms

During the study period 203 cultures of peritoneal dialysis effluent were performed. The microbiological causes of peritonitis are summarized in Table 3. Gram-positive and Gram-negative bacteria accounted for 74.1% and 21.3% of peritonitis, respectively.

**Table 3:** Microbiological causes and efficacy rate of first-line empirical antibiotic therapy in peritoneal dialysis related peritonitis episodes. EAT = empirical antibiotic therapy; ep/pty = number of episodes per patient-year; n = number. *Percentage calculated on the total of isolated bacteria (*n* = 143); **Percentage calculated on the total of accomplished cultures (*n* = 203)

		Organism	ALL *n* = 219, (%**)	Rate (ep/pty)	Efficacy EAT (%)
	n (%*)				
		Streptococcus species	37 (18.2)	0.05	34 (92)
		Staphylococcus aureus	24 (11.8)	0.03	15 (60)
		Staphylococcus epidermidis	17 (8.4)	0.02	14 (82.4)
GRAM +	106 (74.1)	Staphylococcus other species	6 (3)	0.007	5 (83.3)
		Enterococcus faecalis	12 (5.9)	0.02	7 (58.3)
		Enterococcus faecium	4 (2)	0.005	3 (75)
		Micrococcus luteus	3 (1.5)	0.004	2 (66.7)
		Corinebacterium species	3 (1.5)	0.004	1 (33.3)
		Escherichia coli	18 (8.9)	0.02	2 (11.1)
		Pseudomonas aeruginosa	4 (2)	0.005	1 (25)
		Klebsiella Pneumoniae	4 (2)	0.005	2 (50)
		Acinetobacter species	3 (1.4)	0.004	2 (66.7)
		Stenothropomonas species	2 (1)	0.002	2 (100.0)
GRAM -	37 (25.9)	Serratia marcescens	1 (0.5)	0.001	0 (0)
		Proteus mirabilis	1 (0.5)	0.001	0 (0)
		Morganella morganii	1 (0.5)	0.001	1 (100)
		Citrobacter freundi	1 (0.5)	0.001	1 (100)
		Enterobacter aerogenes	1 (0.5)	0.001	1 (100)
		Moraxella catarrhalis	1 (0.5)	0.001	1 (100.0)
		Candida albicans	2 (1.0)	0.002	0 (0.0)
FUNGI		Candida parapsilosis	1 (0.5)	0.001	0 (0.0)
		Candida glabrata	1 (0.5)	0.001	0 (0.0)
		Polymicrobial	4 (2)	0.005	0 (0.0)
OTHERS		Negative	52 (25.6)	0.06	37 (71.2)
		Non Accomplished	16	0.02	10 (62.5)

**Table 4:** Microbiological causes and efficacy rate of first-line empirical antibiotic therapy in relapsing, recurrent, and repeat peritonitis episodes. EAT = empirical antibiotic therapy; Eff = efficacy; n = number. *Percentage calculated on the total of isolated bacteria (*n* = 39); **Percentage calculated on the total of accomplished cultures (*n* = 43)

		Organism	ALL *n* = 43, (%**)	Eff EAT (%)	RELAPSING *n* = 19	RECURRENT *n* = 9	REPEAT *n* = 15
	n (%*)						
		Streptococcus species	8 (18.6)	8 (100)	5	0	3
		Staphylococcus aureus	8 (18.6)	6 (75)	4	0	4
		Staphylococcus epidermidis	2 (4.7)	2 (100)	0	1	1
GRAM +	34 (87.2)	Staphylococcus other species	2 (4.7)	2 (100)	1	0	1
		Enterococcus faecalis	6 (14.0)	1 (16.7)	3	1	2
		Enterococcus faecium	2 (4.6)	2 (100)	1	1	0
		Corinebacterium species	4 (9.3)	2 (50)	0	2	2
		Micrococcus luteus	2 (4.6)	1 (50)	2	0	0
		Escherichia coli	3 (7)	0 (0)	0	2	1
GRAM -	5 (12.8)	Acinetobacter Iwoffii	1 (2.3)	0 (0)	1	0	0
		Morganella morganii	1 (2.3)	1 (100)	1	0	0
		Candida albicans	1 (2.3)	0 (0)	0	1	0
		Candida parapsilosis	1 (2.3)	0 (0)	0	1	0
		Negative	2 (4.7)	1 (50)	1	0	1

The two most common Gram-positive microorganisms were Staphylococcus species (23.1%) and Streptococcus species (18.2%), while the most common Gram-negative microorganism was Escherichia coli (8.9%) followed by Pseudomonas aeruginosa (2%) and Klebsiella pneumoniae (2%). Fungal infections were observed in 2% of episodes as frequent as polymicrobial cases (2%).

Culture-negative peritonitis accounted for 25.6% of all episodes (52/203), while in 16 cases the results of culture were missing, thus the causative organism remained unknown. Initial antibiotic regimen was more effective in Gram-positive (76.4%) than Gram-negative (35.1%) microorganisms (*p* < 0.0001). The microbiological causes of relapsing, repeat and recurrent peritonitis are reported in Table 4.

### Incidence and clinical outcomes of cause- and time-specific peritonitis

Rates of relapsing, repeat and recurrent peritonitis were 0.023, 0.018 and 0.011 episode/patient-year, respectively. Altogether they represented approximately 20% of all episodes.

The composite outcome of either catheter removal or death due to peritonitis was higher in relapsing (42.1%, 8/19, *p* < 0.05) and recurrent (44.4%, 4/9, *p* < 0.05) episodes than generic peritonitis (19.1%, 34/178) but similar to repeat peritonitis (3/15, 20%, p = ns).

In comparison with positive-culture peritonitis, culture-negative episodes showed a higher medical cure rate (88.4%, 46/52 vs. 68.9%, 104/151; *p* < 0.01) and were significantly less likely to be complicated by hospitalization (48.1%, 25/52 vs. 72.2%, 109/151; *p* < 0.05), catheter removal (9.6%, 5/52 vs. 22.5%, 34/151; *p* < 0.05), HD transfer (9.6%, 5/52 vs. 21.9%, 33/152; *p* < 0.05) and death (0%, 0/52 vs. 8.6%, 13/151, *p* < 0.05). However, initial antibiotic regimen response was similar between culture-negative and culture-positive episodes (71.2%, 37/52 vs. 62.3%, 94/151, p = ns). As far as catheter-related peritonitis is concerned, 9 episodes of peritonitis in conjunction either with exit-site or tunnel infection were observed. They accounted for 4.2% of all peritonitis and required catheter removal in 77.8% of cases (7/9).

Although peritonitis from enteric causes represented only 2.7% of all episodes, they needed catheter removal in one-third of events (33.3%, 2/6) and caused patient death (66.6%, 4/6) in the remaining cases. Pre-PD peritonitis accounted for 0.5%, while PD catheter insertion-related peritonitis for 2.1% of all episodes.

## Discussion

Abiding by the recent definitions of outcomes and measurement methods [8], our experience confirms that peritonitis still represents a serious cause of morbidity and mortality in PD patients. In fact, peritonitis required hospitalization in more than 50% of the cases and accounted for approximately 35% of the transfer to HD and 7% of deaths.

Our overall peritonitis rate was far below the threshold of 0.4 episode/patient-year proposed by the ISPD recommendations [8]. Furthermore, nearly 60% of the incident patients never experienced peritonitis, while the peritonitis-free survival at 3 months and at one year of treatment was above 90 and 80%, respectively. Those good results might be associated with the early start of in-hospital PD training (mainly the day after the catheter insertion) that patients received in our center [9, 12].

More than two-thirds of the episodes were successfully treated by antibiotic therapy, conversely catheter removal was necessary in less than one-fifth of the events. Those results should reassure PD candidates that even if peritonitis may represent a severe complication, in most of the cases it is manageable by medical treatment.

The empiric antibiotic therapy (first-generation cephalosporin plus aminoglycosides) resulted effective in almost 65% of the cases. Although the initial treatment was successful in the most cases of peritonitis caused by gram-positive bacteria, a second-line antimicrobial therapy was required in almost two-thirds of the episodes due to gram-negative microorganisms. These results drew our attention to act soon on: [1] an investigation regarding Gram-negative antibiotic sensitivities at our center [2], a tobramycin pharmacokinetic study in CAPD.

Whenever empiric antibiotic treatment fails, the identification of the organism and its antibiotic sensitivities indicate the possible source of infection and help physicians to guide the choice of therapy. In our experience, the rate of culture-negative peritonitis was approximately 25%, substantially greater than the suggested ISPD benchmark of 15% [8].

Infectious culture-negative peritonitis may result from recent antibiotic exposure, suboptimal sample collection or culture methods, or unusual organisms.

Thus, we promptly revised the method of culturing PD effluent aiming at enhancing the yield of peritoneal fluid culture and reducing the time needed for a positive result. Two significant changes that we recently implemented were the following: [1] centrifugation of 50 ml of PD effluent followed by resuspension of the sediment in 5 ml and subsequent inoculation in blood-culture bottles; [2] routinely culture of PD dialysate in both aerobic and anaerobic media.

Culture-negative peritonitis compared with positive-culture episodes showed a more benign outcome in terms of hospitalization, catheter removal, HD transfer and death. Our results are in accordance with those of the large report from Australia and New Zealand Dialysis and Transplant Registry [13], while are in contrast with Szeto’s data [14]. However, in the latter study only a low proportion of patients (< 5%) received the antibiotics at the onset of culture-negative peritonitis, while there was a significative delay in the timely administration of antimicrobial therapy in most of the patients, suggesting that if antibiotic treatment is promptly initiated, culture-negative peritonitis may likely show more favorable clinical outcomes than culture-positive episodes.

Furthermore, nearly all patients that required catheter removal as a rescue treatment for peritonitis, were permanently transferred to HD. Thus, the development of strategies aimed either at maintaining patients or timely shifting them back to PD are urgently needed. The use of simultaneous removal and replacement of peritoneal catheter in relapsing peritonitis after resolution of clinical signs and normalization of effluent WBC count has been proposed [15, 15]. A similar approach could be also implemented in a case of a refractory peritonitis if it shows a partial response to the antibiotic therapy [17]. In the remaining cases, whenever feasible, the replacement of the PD catheter should be advocated as soon as possible to avoid long transit on HD [18].

In our center pre-PD peritonitis percentage was extremely low (less than 1%) due to the double purse-string used for catheter placement that in most cases allowed the patient to initiate the peritoneal exchanges within 24 h from catheter insertion [9].

Cause-specific peritonitis that carry an inauspicious prognosis are enteric and catheter-associated peritonitis. We recorded a relatively low rate of those two entities in comparison to other series. As far as catheter-related peritonitis is concerned, in our unit the extensive use of mini-invasive surgical technique in refractory tunnel infections could have prevented the occurrence of secondary peritonitis in some cases [19, 20]; while the employ of simultaneous removal and replacement of peritoneal catheter in peritonitis arising in conjunction with the exit-site or tunnel infections could have decrease the associated HD transfer rate [21].

The outcome of enteric peritonitis has not changed despite the many improvements in the practice of PD [22, 23]. In our series these episodes were associated with death in above 60% of the patients. It is possible that early diagnosis of surgical peritonitis and exploratory laparotomy might improve outcomes [23]. Thus, in case of high suspicion of enteric peritonitis and no improvement after 48 h from catheter removal together with broad-spectrum antibiotic therapy (including coverage for anaerobic bacteria), we are now aiming at carrying out a fast surgical referral to promptly evaluate the need of either a diagnostic laparoscopy or an exploratory laparotomy.

Considered altogether, relapsing, recurrent and repeat peritonitis accounted for approximately 20% of all episodes. Previous studies indicated that relapsing and recurrent as well as repeat peritonitis represent distinct clinical entities [24–26].

Notably, our data confirmed that compared with generic episode, repeat peritonitis do not possess a higher rate of complications. Conversely, relapsing and recurrent peritonitis were significantly more likely to be complicated by catheter removal and death showing approximately a double probability. Thus, the causes of these two entities should be deeply investigated in the future, aiming at devising adequate preventive measures to decrease the associated-morbidity and mortality.

The limitations of our study are mainly related to its single-center and retrospective nature. However, in comparison with registry studies, these data should be more complete, and their accuracy easier to be verified.

Despite the previous drawbacks, the paper includes long-term follow-up of a relatively large and unselected PD cohort with data collection performed trough the support of an electronic database. In summary, our study adds additional evidence to some relevant aspects concerning PD-associated peritonitis. Although representing the main cause of HD transfer and a potentially severe complication, more than 50% of incident PD patients will never experience peritonitis. Furthermore, entities such as relapsing, recurrent and enteric peritonitis carry a worse outcome than generic peritonitis. Thus, these episodes should be promptly addressed and aggressively treated. Conversely, culture-negative peritonitis showed a better prognosis as compared to positive-culture episodes. However, aiming at the prevention of microbial resistances, the reduction of culture-negative cases is necessary to minimize patient exposure to empiric therapy with multiple antibiotics. In our experience first-line empirical antibiotic therapy, constituted by intraperitoneal cefazolin in association with tobramycin, was effective in almost 65% of the cases. Nevertheless, the efficacy of this regimen was unsatisfactory in Gram-negative microorganisms requiring further microbiological sensitivities investigations as well as pharmacokinetic evaluation.

In conclusion, the use of standardized definitions followed by a defined protocols for peritonitis treatment based on the sources of the bacteria involved may enhance clinical practice and allows comparative studies.

## Data Availability

The datasets used and/or analysed during the current study available from the corresponding author on reasonable request.
